# Involvement of the Sch9/Rim15/Msn2 signaling pathway in the anti-aging activity of dendrobine from *Dendrobium nobile* Lindl. via modification of oxidative stress and autophagy

**DOI:** 10.1186/s13020-023-00827-4

**Published:** 2023-09-05

**Authors:** Enchan Wu, Yiting Lian, Sali Zhao, Yajing Li, Lan Xiang, Jianhua Qi

**Affiliations:** College of Pharmaceutical Sciences, Zhejiang University, Yu Hang Tang Road 866, Hangzhou, 310058 China

**Keywords:** Dendrobine, *Dendrobium nobile* Lindl., Anti-aging, Oxidative stress, Autophagy, Sch9/Rim15/Msn2 signaling pathway

## Abstract

**Background:**

Aging is an important pathogenic factor of age-related diseases and has brought huge health threat and economic burden to the society. *Dendrobium nobile* Lindl., a valuable herb in China, promotes longevity according to the record of ancient Chinese materia medica. This study aimed to discover the material basis of *D. nobile* as an anti-aging herb and elucidate its action mechanism.

**Methods:**

K6001 yeast replicative lifespan assay was used to guide the isolation of *D. nobile.* The chronological lifespan assay of YOM36 yeast was further conducted to confirm the anti-aging activity of dendrobine. The mechanism in which dendrobine exerts anti-aging effect was determined by conducting anti-oxidative stress assay, quantitative real-time PCR, Western blot, measurements of anti-oxidant enzymes activities, determination of nuclear translocation of Rim15 and Msn2, and replicative lifespan assays of Δ*sod1*, Δ*sod2*, Δ*cat*, Δ*gpx*, Δ*atg2*, Δ*atg32*, and Δ*rim15* yeasts.

**Results:**

Under the guidance of K6001 yeast replicative lifespan system, dendrobine with anti-aging effect was isolated from *D. nobile*. The replicative and chronological lifespans of yeast were extended upon dendrobine treatment. In the study of action mechanism, dendrobine improved the survival rate of yeast under oxidative stress, decreased the levels of reactive oxygen species and malondialdehyde, and enhanced the enzyme activities and gene expression of superoxide dismutase and catalase, but it failed to elongate the replicative lifespans of Δ*sod1*, Δ*sod2*, Δ*cat*, and Δ*gpx* yeast mutants. Meanwhile, dendrobine enhanced autophagy occurrence in yeast but had no effect on the replicative lifespans of Δ*atg2* and Δ*atg32* yeast mutants. Moreover, the inhibition of Sch9 phosphorylation and the promotion of nuclear translocation of Rim15 and Msn2 were observed after treatment with denrobine. However, the effect of dendrobine disappeared from the Δ*rim15* yeast mutant after lifespan extension, oxidative stress reduction, and autophagy enhancement.

**Conclusions:**

Dendrobine exerts anti-aging activity in yeast via the modification of oxidative stress and autophagy through the Sch9/Rim15/Msn2 signaling pathway. Our work provides a scientific basis for the exploitation of *D. nobile* as an anti-aging herb.

**Supplementary Information:**

The online version contains supplementary material available at 10.1186/s13020-023-00827-4.

## Background

Aging is an inevitable and progressive process of organisms, which is manifested as the gradual decline in physiological functions and change in psychological activities. Aging is an important pathogenic factor for cancer, cardiovascular disease, Alzheimer’s disease, and other age-related diseases [1]. With the soaring number of globally aged population, the medical expenses caused by age-related diseases bring huge economic burdens to the society and governments [2, 3]. Therefore, delaying aging and achieving healthy aging are important for the development of individuals and society. At present, pharmaceutical intervention by anti-aging molecules has anti-aging effects, and various molecules such as resveratrol (RES), rapamycin (RA), and dasatinib, a kind of senolytics which postpone aging by inducing death of senescent cells, have shown their potential to delay aging [1]. However, none of drugs in the market are used to slow down aging. Accordingly, the development of anti-aging medicine is still an urgent problem for researchers.

Budding yeast *Saccharomyces cerevisiae* is a commonly used model organism to study aging. Compared with mammals, this species presents the advantages of low cost, short life cycle, and easier genetic manipulation [4]. Yeasts have two different lifespans, namely, chronological and replicative lifespans. Chronological lifespan is defined as the length of time non-dividing yeast cells that survive in a medium. Replicative lifespan denotes the number of daughter cells that a single mother cell reproduces before death. The traditional assay of replicative lifespan is inconvenient and time-consuming because of complex microdissection [5]. K6001 strain is a genetic mutant yeast derived from W303, in which only mother cells could reproduce offspring in glucose medium and not the daughter cells [6]. Consequently, the assay of reproductive lifespan becomes rapid and high-throughput when K6001 yeast is applied. In this study, the replicative lifespan assay of K6001 strain was utilized to screen molecules with anti-aging activity from *Dendrobium nobile* Lindl., which is a famous herb in China.

Reactive oxygen species (ROS) refers to a series of highly reactive oxygen-containing radicals, resulting from the incomplete reduction of oxygen during aerobic metabolism. ROS plays a vital role in signal transduction and resistance to pathogen invasion [7]. However, excessive ROS attacks biological macromolecules such as DNA, lipids, and proteins [7], causing oxidative damages in cells and tissues. Under normal physiological conditions, excess ROS is eliminated by the endogenous antioxidant system such as superoxide dismutase (SOD), catalase (CAT), and glutathione peroxidase (GPx) to maintain homeostasis [8]. Through aging, this balance is continually disrupted, exposing the organisms to chronic oxidative stress, which is considered as the limiting factor of lifespan. Moreover, the overexpression of MnSOD could extend the lifespan of adult *Drosophila* [9]. Mice with mitochondrial overexpression of catalase exhibited a reduction of oxidative damages, mitochondria deletion development, and prolonged lifespan [10]. So far, enhancing the activity of antioxidant enzymes is one of crucial mechanism for anti-aging compounds discovered in previous studies [11, 12].

Autophagy is a highly conserved physiological process, which degrades damaged or faulty proteins, organelles, and even invasive microbes [13], because autophagy allows the cells to maintain proteostasis and normal functioning. Autophagy is closely linked to aging. A decline in autophagy capacity is observed in animal models with aging [14], and the genetic manipulation that increases autophagy extends lifespan [15]. Additionally, some anti-aging molecules exert lifespan-extending activity via autophagy [11, 12]. To date, approximately 18 genes of autophagy-related (*ATG*) genes are important in kinds of autophagy, as the encoding proteins of these *ATG* genes are essential in autophagosome formation, and these proteins are denoted as core Atg protein [13]. Atg8 is a core Atg protein, which mediates the phagophore expansion [13]. Here, YOM38 strain, which expresses GFP-Atg8 fusion protein, was used to detect autophagy occurrence, because GFP-Atg8 is cleaved by vacuolar hydrolases during autophagy and releases the relatively stable GFP [16]. *ATG2* and *ATG32* are two important *ATG* genes that encode Atg2 or Atg32, respectively. Atg2 interacts with Atg18 to assist Atg9 in the membrane delivery of expanding phagophore [13]. Atg32 is specifically involved in mitophagy and functions as a receptor in the selective recognition of mitochondria [17].

The target of rapamycin complex 1 (TORC1) plays a central role in the network of regulation of cell growth and metabolism by sensing environmental changes [18]. The inhibition of TORC1 signaling pathway leads to the longevity of yeasts, worms, flies, and rodents [19]. In yeast, Sch9 is one of downstream effectors of TORC1, and Rim15 mediates the lifespan-prolonging effect of the TORC1/Sch9 signaling pathway [20]. The inhibition of TORC1 results in the dephosphorylation of Sch9 followed by nuclear translocation of Rim15 [18, 21]. The Rim15 in nucleus directly phosphorylates the downstream effectors Msn2/4 [18], which are two homologous transcription factors that regulate the transcription of genes containing the stress response element in their promoter region such as *SOD2* [18, 22]. Additionally, the Rim15-dependent transcription of genes is involved in stress response and oxidative stress response [23]. Nuclear translocation of Rim15 from cytoplasm leads to the inhibition of Ume6, thus inducing *ATG8* and enhancing autophagy [24].

*D. nobile* Lindl., a valuable traditional Chinese herb, can be used for the treatment of stomach diseases and promote longevity in ancient Chinese materia medica. In the past decades, this plant has been used for the treatment of cancers, hyperlipidemia, and hyperglycemia, which are considered as age-related diseases [25]. *D. nobile* contains alkaloids, sesquiterpenes, polysaccharides, bibenzyl, and other chemical components and exerts various pharmacological activities such as antioxidant, anti-tumor, and immune enhancement [26]. In addition, the polysaccharide from *D. nobile* has been indicated to reduce the blood glucose and ameliorate the testicular damage in diabetic rats [27]. The total alkaloids of *D. nobile* improved cognitive dysfunction in the animal models of Alzheimer’s disease by regulating tau protein hyperphosphorylation, activating autophagy, and inhibiting neuronal apoptosis and neuroinflammation [28]. In the present study, an anti-aging compound named dendrobine was isolated from *D. nobile* under the guidance of replicative lifespan assay of yeast, and the action mechanism of dendrobine was explored by utilizing biology techniques.

## Methods

### General and yeast strains

Analytical pure reagents (methanol and dichloromethane from Sinopharm Chemical Reagent Co. Ltd., Shanghai, China), triethylamine and HPLC-grade MeCN (J&K Scientific Ltd., Beijing, China), silica gel (200–300 mesh, Yantai Research Institute of Chemical Industry, Yantai, China) and Cosmosil 5C18-MS-II packed column (Nacalai Tesque, Japan) were used for the isolation and purification of natural products. TLC silica gel plates (Yantai Jiangyou Silicone Development Co., Ltd., Yantai, China) were utilized for thin-layer chromatography analysis. CDCl_3_ (Cambridge Isotope Laboratories, Inc., Andover, WA, USA) was used as the solvent for ^1^H NMR. ^1^H NMR spectra and HR ESI-TOF-MS data were obtained using a Bruker AV III-500 spectrometer (Bruker, Karlsruhe, Germany) and Agilent 6224A LC/MS (Agilent Technologies Inc., Beijing, China), respectively. The following reagents and compounds were purchased from the indicated manufacturer and used in biological experiments: *n*-butanol, chloroform, and isopropanol (Sinopharm Chemical Reagent Co., Ltd., Shanghai, China), dimethyl sulfoxide (DMSO) (Sigma, Saint Louis, MO, USA), 4’,6’-diamidino-2-phenylindole (DAPI) dihydrochloride and Hoechst 33,342 (Macklin, Shanghai, China), RES (J&K Scientific Ltd., Beijing, China), rapamycin (Solarbio, Beijing, China), and wortmannin (Beyotime Biotech, Shanghai, China). Ethanol was used as solvent to dissolve the compounds or as negative control in experiments for yeasts. DMSO was used as solvent to dissolve the compounds or as negative control in the assay system of PC12 cells.

K6001 yeast derived from W303 was provided by Professor Michael Breitenbach (University of Salzburg, Austria). BY4741, BY4741 expressing sfGFP-Sch9-5HA, Rim15-GFP or Msn2-GFP, Δ*sod1*, Δ*sod2*, Δ*cat*, Δ*gpx*, Δ*atg2*, Δ*atg32*, and Δ*rim15* of K6001, YOM36, and YOM38 containing pRS316-*GFP-ATG8* plasmid were provided by Professor Akira Matsuura (Chiba University, Japan). The genotypes of the above yeast strains are presented in Additional file 1: Table S1.

### Isolation and purification of dendrobine

**Fig. 1:**
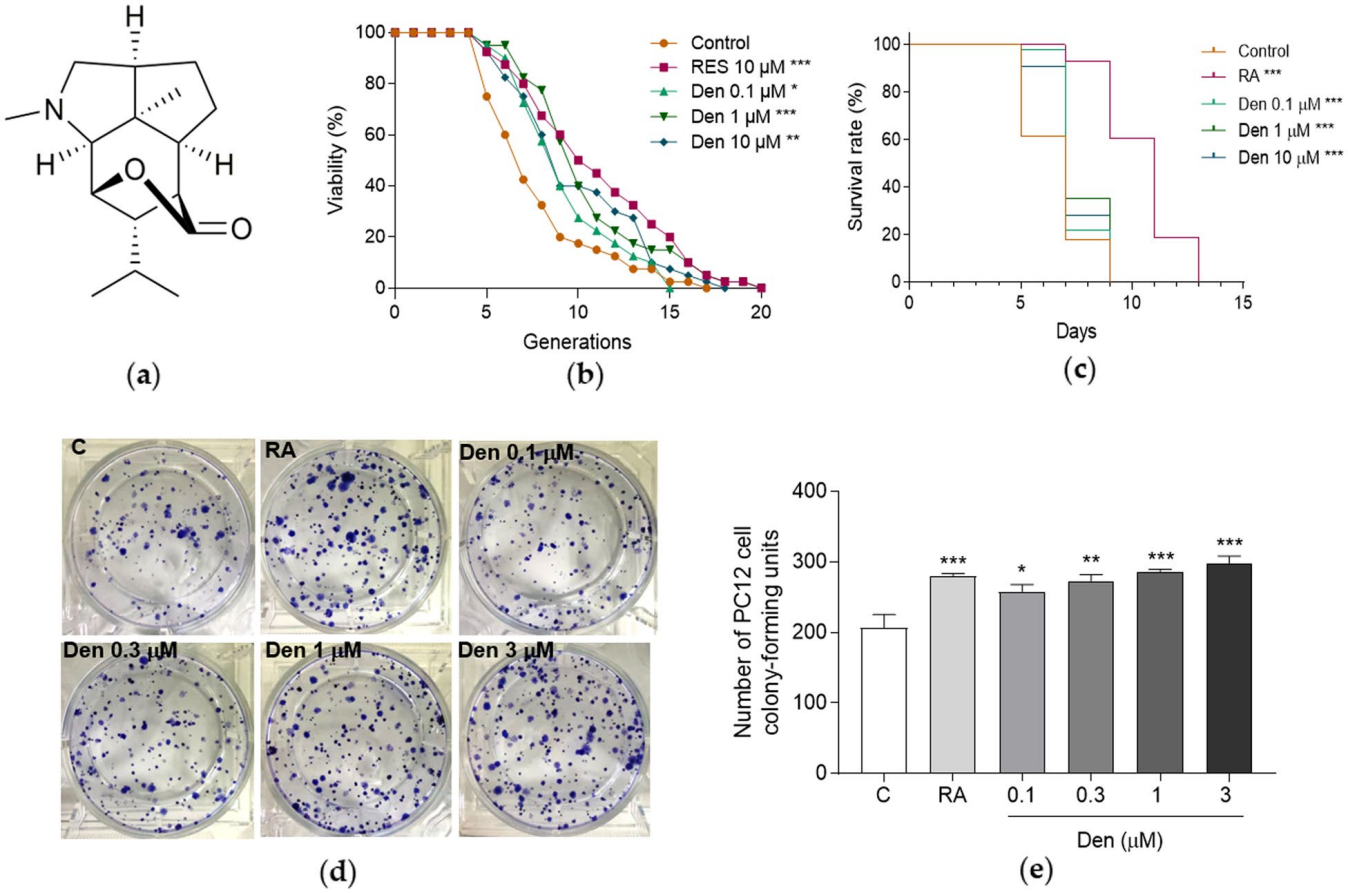
The chemical structure and anti-aging activity of dendrobine. **A** The chemical structure of dendrobine. **B** Replicative lifespan-prolonging effect of dendrobine on K6001 yeast. **C** Effect of dendrobine on chronological lifespan in YOM36 yeast. **D** The photograph of colonies of PC12 cells after treating with different concentrations of dendrobine. **E** The digital result of **D**. The experiment was repeated three times and the data from each experiment are displayed as mean ± SEM. The repeats number of each group for replicative lifespan assay was 40, and the repeats number of each group for chorological lifespan assay was five. ^*^, ^**^ and ^***^ represent significant difference compared with negative control (*p* < 0.05, *p* < 0.01, *p* < 0.001)

*D. nobile* was obtained from Xintian Hu Mantang Pharmaceutical Co., Ltd. (Chishui, Guizhou, China). A voucher specimen (no. 20,220,723) was preserved in Zhejiang University, Institute of Materia Medica. Fresh stems of *D. nobile* (375 g) were crushed and extracted with 2 L of methanol for 24 h (repeated once). Then, the supernatant was concentrated under vacuum to obtain a crude methanol extract (15.0 g). The crude methanol extract was chromatographed on a silica gel column eluted with CH_2_Cl_2_/MeOH (100:0, 100:1, 80:1, 40:1, 20:1, 10:1, 1:1, 0:100). The most active fractions (78.1 mg) eluted by CH_2_Cl_2_/MeOH (20:1) were purified by HPLC (Cosmosil 5C18-MS-II packed column [10 × 250 mm]; MeCN/0.1% triethylamine in water: 30:70–80:20 in a linear gradient in 30 min; flow rate: 3 mL/min; detection wavelength: 210 nm) to afford an active sample. This active sample was subjected to HPLC for further purification (Cosmosil 5C18-MS-II packed column [10 × 250 mm]; MeCN/0.1% triethylamine in water: 50:50; flow rate: 3 mL/min; detection wavelength: 210 nm), and an active molecule (3.8 mg, t_R_= 15 min) was obtained. The molecule was identified as dendrobine by comparing the ^1^H NMR and MS spectra with previous literature [29]. HR ESI-TOF-MS *m/z* [M + H]^+^ 264.1946, which was calculated for C_16_H_26_NO_2_ [M + H]^+^ 264.1958. ^1^H NMR (500 MHz, CDCl_3_): ^1^H NMR (500 MHz, CDCl_3_): *δ* = 4.84 (1H, dd, *J* = 5.5, 3.0 Hz), 3.20 (1H, t, *J* = 8.7 Hz), 2.72 (1H, s), 2.70 (1H, d, *J* = 8.7 Hz), 2.53 (3H, s), 2.46 (1H, dd, *J* = 5.5, 4.4 Hz), 2.39 (1H, quint, *J* = 8.4 Hz), 2.13 (1H, m), 2.10 (1H, m), 2.06 (1H, m), 2.02 (1H, m), 1.87 (1H, m), 1.79 (1H, m), 1.55 (1H, m), 1.40 (3H, s), 0.98 (3H, d, *J* = 2.7 Hz), 0.97 (3H, d, *J* = 2.7 Hz). The ^1^H NMR spectrum is presented in Additional file 1: Fig. S1. The structure of dendrobine is shown in Fig. 1A.

### Replicative and chronological lifespan assay

The replicative lifespan assays were conducted according to a previous methodology [11]. In brief, the K6001 yeast cryopreserved at − 30 °C was inoculated in galactose liquid medium (3% galactose, 2% peptone, 1% yeast extract) and cultured at 28 °C for 24 h. A quantity of yeasts was transferred into a centrifuge tube and washed by PBS for three times. Afterward, the yeasts were counted, and approximately 4,000 yeasts were spread evenly on YPD (1% yeast extract, 2% hipolypeptone, 2% D-glucose) agar plates supplemented with negative control or 10 µM RES or indicated concentrations of samples. After incubation at 28 °C for 48 h, 40 microcolonies were randomly selected under an Olympus upright microscope (Olympus Corporation, Tokyo, Japan), and the number of daughter cells of a mother cell was calculated. The method to assay the replicative lifespans of Δ*sod1*, Δ*sod2*, Δ*cat*, Δ*gpx*, Δ*rim15*, Δ*atg2*, and Δ*atg32* yeast mutants with K6001 background was similar to that of wild-type yeast.

Chronological lifespan assay was carried out in accordance with previous study [11]. YOM36 yeast was inoculated in YPD overnight. The cultivated yeast with initial OD_600_ value of 0.01 was transferred to synthetic defined (SD) medium (0.17% yeast nitrogen base without amino acids and ammonium sulfate, 0.5% ammonium sulfate, and 2% glucose) and treated with 1 µΜ rapamycin or 0, 0.1, 1, or 10 µM dendrobine (denoted as day 0). After incubation for 72 h, 200 yeasts from each group were smeared onto YPD agar plates and incubated for 48 h. The colony-forming units (CFUs) on each plate were counted. This step was repeated every two days until the survival rate (survival rate = CFUs/ CFUs on day 3 × 100%) reached below 5%.

### Y**east-like chronological lifespan assay**

This analysis was conducted according to previous study [12]. Briefly, PC12 cells was recovered in complete medium (CM) which contained 10% premium horse serum (Soleibao Technology Co. Ltd., Beijing, China), 7.5% fetal bovine serum and 1% penicillin–streptomycin solution (CellMax Cell Technology Co. Ltd., Beijing, China) for 7 days. Then, approximately 80,000 PC12 cells were seeded in each well of a 96-well plate and incubated for 24 h. Afterwards, the medium was replaced by serum-free DMEM containing 0.5% DMSO as control, rapamycin at 1 µM as positive control, or dendrobine at doses of 0.1, 0.3, 1 and 3 µM. The cells were cultured, and the medium was replaced by serum-free DMEM containing DMSO and samples every 2 days. After culture for 5 days, the cells were trypsinized, and the 2% cell suspensions were added into six-well plates supplemented with CM. After the cells were continually incubated for 15 days, the colonies of PC12 cells formed on plates were stained with crystal violet and photographed.

### Assay of yeast growth under oxidative stress

BY4741 yeasts with OD_600_ value of 0.1 were cultured in YPD and treated with 10 µΜ RES or 0, 0.1, 1, or 10 µM dendrobine. After shaking at 28 °C for 24 h, 5 µL of each yeast culture with OD_600_ value of 1.5 was dropped onto YPD agar plates (containing 9.5 mM hydrogen peroxide). The growth of yeast in each group was photographed after incubation for 72 h. For quantitative study, BY4741 yeasts were treated with RES at a concentration of 10 µΜ or dendrobine at concentrations of 0, 0.1, 1, or 10 µM. After incubation for 24 h, almost 200 yeasts were spread onto YPD agar plates with or without 5.5 mM hydrogen peroxide. The survival rate of each group was calculated after 48 h of cultivation (survival rate = the number of colonies growing on medium with hydrogen peroxide/the number of colonies growing on medium without hydrogen peroxide × 100%).

### Measurements of ROS and malondialdehyde (MDA) levels

The BY4741 yeast with initial OD_600_ value of 0.1 was cultured in YPD and treated with RES at a dose of 10 µM or dendrobine at doses of 0, 0.1, 1, and 10 µM for 24 or 48 h, respectively. The Δ*rim15* yeast mutant with K6001 background was inoculated in galactose liquid medium overnight. Then, the recovered cells with initial OD_600_ value of 0.1 were transferred to galactose liquid medium supplemented with 10 µM RES or 0, 1 µM dendrobine and cultured for 24 h.

The level of ROS was measured by collecting the yeasts and washing them with PBS for three times. Each group was treated with 2′,7′-dichlorodihydrofluorescein diacetate (DCFH-DA) at final concentration of 10 µM under dark condition with shaking for 1 h. After washing with PBS, the fluorescence intensity of DCF (2′,7′-dichlorofluorescein) of 1 × 10^7^ cells was recorded using a Varioskan® flash spectral scanning multimode reader (Thermo Fisher Scientific, Waltham, MA, USA) at excitation wavelength of 488 nm and emission wavelength of 525 nm.

In the quantification of MDA content, yeasts were collected and washed with PBS. Subsequently, PBS and grinding beads were added. The yeast cells were ground at 70 Hz for 1 min by using an automatic sample fast grinder (Shanghai Jingxin Inc., Shanghai, China). After centrifugation at 12,000 ×g and at 4 °C for 10 min, the supernatant was used as protein sample. Protein concentration was determined using the BCA kit (CoWin Biotech, Beijing, China). The MDA level in yeast was measured using the MDA assay kit (Nanjing Jiancheng Bioengineering Institute, Nanjing, China) according to the manufacturer’s instructions. In brief, the test samples, anhydrous ethanol (negative control), and 10 nmol/L standard were mixed with 100 µL of reagent I. Then, 375 µL of reagent II and 125 µL of reagent III were added. After mixing, each group was heated in water bath at 95 °C for 40 min, taken out, and cooled with running water. Subsequently, 200 µL of supernatant of each sample was added to a 96-well plate after centrifugation for 10 min. Finally, BioTek microplate reader (BioTek, Winooski, VT, USA) was used to measure the A_532_ value of the supernatant.

### Quantification of SOD, CAT, and GPx enzyme activities

The methods for BY4741 yeast culture, protein extraction, and protein concentration determination were the same as the measurement of MDA. The protein concentration of all the protein samples was diluted to 1.25 µg/µL before enzyme activity measurement. The enzyme activities were measured following the manufacturer’s instructions of SOD assay kit (Nanjing Jiancheng Bioengineering Institute, Nanjing, China), CAT assay kit (Beyotime Biotech, Shanghai, China), and GPx assay kit (Beyotime Biotech, Shanghai, China). The detailed procedures are presented in Additional file 1.

### Fluorescence imaging of autophagy

The YOM38 yeasts containing the pRS316-*GFP-ATG8* plasmid were inoculated in YPD overnight. The cultured cells at OD_600_ value of 0.1 were transferred to SD medium supplemented with RES at a dose of 10 µM or dendrobine at doses of 0, 0.1, 1 or 10 µM. After incubation for 22 h, the yeasts were collected and stained by DAPI with final concentration of 20 µg/mL. The dyed yeasts were washed with PBS, suspended in 30% glycerin solution, and imaged using a two-photon confocal fluorescence microscope (Olympus FV1000BX-51, Tokyo, Japan).

For K6001 and Δ*rim15* of K6001 yeast, two kinds of yeasts were recovered in galactose liquid medium overnight respectively. Then, the cultivated yeast with OD_600_ value of 0.1 was cultured in galactose liquid medium and treated with 10 µM RES or 0 and 1 µM dendrobine for 22 h. According to the instructions of the autophagy detection kit (Enzo Life Sciences, New York, NY, USA), the yeasts were washed with PBS and dyed using the green detection reagent in the dark for 1 h. Then, the yeasts were washed thrice and stained with DAPI. The two-photon confocal fluorescence microscope (Olympus FV1000BX-51, Tokyo, Japan) was utilized to visualize autophagy flux in yeast.

### Visualization of protein nuclear translocation

For Msn2-GFP nuclear translocation experiment, BY4741 yeast expressing Msn2-GFP was grown to early log phase in YPD. Subsequently, the yeasts were treated with 1 µΜ rapamycin and 0, 0.1, 1, 10 µM dendrobine for 2 h. After collecting and washing the yeasts, they were stained with DAPI at final concentration of 20 µg/mL and suspended in 30% glycerin solution. The two-photon confocal fluorescence microscope (Olympus FV1000BX-51, Tokyo, Japan) was utilized to image Msn2-GFP.

In the nuclear translocation of Rim15-GFP, BY4741 yeast expressing Rim15-GFP was inoculated in YPD and shaken for 3 days. After treatment with 1 µΜ rapamycin and 0, 0.1, 1, and 10 µM dendrobine for 6 h, the yeasts were collected and dyed with Hoechst 33,342 (final concentration was 1 µg/mL). The fluorescence of yeasts was recorded using Olympus BX61 fully motorized upright fluorescence microscope (Olympus, Tokyo, Japan).

### RT-PCR analysis

BY4741 yeasts were cultured in YPD for 24 or 48 h and treated with 10 µM RES or 0, 0.1, 1, and 10 µM dendrobine. The yeasts were collected, and grinding beads equal volume to yeast and TRIzon (CoWin Biotech, Beijing, China) were added. Then, the mixture was ground at 68 Hz by using an automatic sample fast grinder (Shanghai Jingxin Inc., Shanghai, China) for 3 min and stood on ice for 10 min. Subsequently, 200 µL of chloroform was added, and the mixture was vortexed for 1 min and stood on ice for 5 min. After centrifugation (12,000 ×g, 4 °C, 15 min), isopropanol was added to the colorless aqueous phase, and the mixture of isopropanol and colorless aqueous phase was mixed softly and stood on ice for 10 min. After centrifugation (12,000 ×g, 4 °C) for 10 min, 75% ethanol was added to wash the pellet twice, and the pellet containing the RNA was dissolved in RNase free water. The concentration of RNA was measured using Eppendorf Biophotometer Plus (Eppendorf Company, Hamburg, Germany). Approximately 5 µg of RNA was reverse-transcribed to synthesize cDNA by employing the HiFi-MMLV cDNA Kit (CoWin Biotech, Beijing, China). The mRNA abundance of each group was determined by qRT-PCR method by using SYBR Premix EX Taq™ (Takara, Otsu, Japan) and CFX96 Touch (Bio-Rad, Hercules, USA). The primer sequence used are as follows: *SOD1*, sense 5′-CAC CAT TTT CGT CCG TCT TT-3′ and antisense 5′-TGG TTG TGT CTC TGC TGG TC-3′; *SOD2*, sense 5′-CTC CGG TCA AAT CAA CGA AT-3′ and antisense 5′-CCT TGG CCA GAA GAT CTG AG-3′; *CAT*, sense 5′-TGA CAA ACT CCA CTG GTA ATC C-3′ and antisense 5′-TCC CTG TTG AAA TGA GCC AA-3′; *GPx*, sense 5′-CGC TCC GTC AAG TAA ACA TAG G-3′ and antisense 5′ -GGC CGC TGT TAT TGT TTT GAA C-3′; and *TUB1*, sense 5′-CCA AGG GCT ATT TAC GTG GA-3′ and antisense 5′-GGT GTA ATG GCC TCT TGC AT-3′. The thermal recycling parameters were as follows: *SOD1* and *SOD2*, 95 °C for 2 min, followed by 40 cycles, 95 °C for 15 s, 55 °C for 25 s, and 72 °C for 10 s; *GPx* and *CAT*, 40 cycles, 95 °C for 15 s, 56 °C for 35 s. The 2^−ΔΔCt^ method was used to analyze the raw data obtained from qRT-PCR. The mRNA abundance of each gene was normalized to those of *TUB*1.

### Western blot

For the quantification of autophagic level, the reinvigorated YOM38 yeast containing the pRS316-*GFP-ATG*8 plasmid was inoculated in SD medium with yeast at an initial OD_600_ value of 0.1 and treated with 300 µM RES, 0, 0.1, 1, and 10 µM dendrobine, or 200 nM wortmannin for 22 h. During the course of experiment, YOM38 yeasts at OD_600_ value of 0.1 were cultured in SD medium. The yeasts treated with 1 µM dendrobine were collected at 0, 8, 15, and 22 h, while the groups treated with 300 µM RES or 200 nM wortmannin plus 1 µM dendrobine were collected at 22 h. In the determination of sfGFP-Sch9-5HA protein level, the BY4741 yeast expressing sfGFP-Sch9-5HA was grown to the log phase in YPD and then treated with 1 µΜ of rapamycin and 0, 0.1, 1, and 10 µM dendrobine for 40 min. Afterward, the yeasts cells were collected and washed with PBS. Subsequently, grinding beads and PBS supplemented with protease inhibitor cocktail (CoWin Biotech, Beijing, China) and phosphatase inhibitor cocktail (Abcam, Waltham, MA, USA) were added, and the yeasts were ground at 70 Hz for 1 min by using an automatic sample fast grinder (Shanghai Jingxin Inc., Shanghai, China). The cell lysate was centrifuged at 12,000 g and at 4 °C for 10 min, and the supernatant was used as protein sample. Protein concentration was determined using the BCA kit (CoWin Biotech, Beijing, China).

Approximately 30 µg protein of each sample was separated by sodium dodecyl sulfate polyacrylamide gel electrophoresis and then transferred to polyvinylidene fluoride membranes (PVDF, Bio-Rad Laboratones Inc., Hercules, CA, USA). The PVDF membranes were incubated with the primary antibodies of anti-GFP (#598, 1:1000, Medical & Biological Laboratories, Nagoya, Japan), anti-HA (16B12, 1:1000, BioLegend, San Diego, CA, USA), or anti-*β*-actin (#CW0096, 1:1500, CoWin Biotech, Beijing, China; #db6010, 1:1500, Diagbio Ltd., Hangzhou, China). Subsequently, the membranes were washed with PBS with Tween 20 (PBST) and incubated with the secondary antibodies of horseradish peroxidase-linked goat anti-rabbit IgGs (#CW0103, 1:5000, CoWin Biotech, Beijing, China) for GFP or goat anti-mouse IgGs (#CW0102, 1:5000, CoWin Biotech, Beijing, China) for sfGFP-Sch9-5HA and *β*-actin for 40 min. After washing with PBST, the bands of protein on the membrane were visualized using the e-ECL Western blot kit (CoWin Biotech, Beijing, China), and ImageJ software (National Institute of Health, Rockville, MD, USA) was used for band digitization.

### Statistical analysis

The 8.0.2 vision software of GraphPad Prism software (GraphPad Prism, San Diego, CA, USA) was used for statistical analysis. Ordinary one-way ANOVA followed by Dunnett’s multiple comparisons test was used to evaluate statistically significant differences among groups. Two-tailed, unpaired Student’s t-tests were used for comparison between two groups. The chronological lifespan assay of the yeast was analyzed using the log-rank (Mantel-Cox) test. Statistical significance was considered at *p* < 0.05.

## Results

### Dendrobine extends yeast lifespan

Dendrobine, a sesquiterpenoid alkaloid, was isolated and purified from the methanol extracts of *D. nobile* under the guidance of assay system of K6001 replicative lifespan. As presented in Fig. 1B and Additional file 1: Table S2, the replicative lifespan of K6001 yeast was significantly extended by dendrobine at concentrations of 0.1, 1 and 10 µM (*p* < 0.05, *p* < 0.001 and *p* < 0.01, respectively).

The anti-aging activity of dendrobine was confirmed using YOM36 yeast to conduct the chronological lifespan assay. Dendrobine at doses of 0.1 1, and 10 µM clearly improved the survival rates of yeast compared with negative control (Fig. 1C, *p* < 0.001, *p* < 0.001 and *p* < 0.001, respectively). Furthermore, we performed yeast-like chorological lifespan assay in PC12 cells. Dendrobine clearly improved the survival rates of PC12 cells compared with negative control (Fig. 1D, E, *p* < 0.05*, p* < 0.01, *p* < 0.001 and *p* < 0.001, respectively). These data suggest that dendrobine exhibits anti-aging activity for yeast and mammal cells.

### Dendrobine increased the resistance of yeast to oxidative stress

**Fig. 2:**
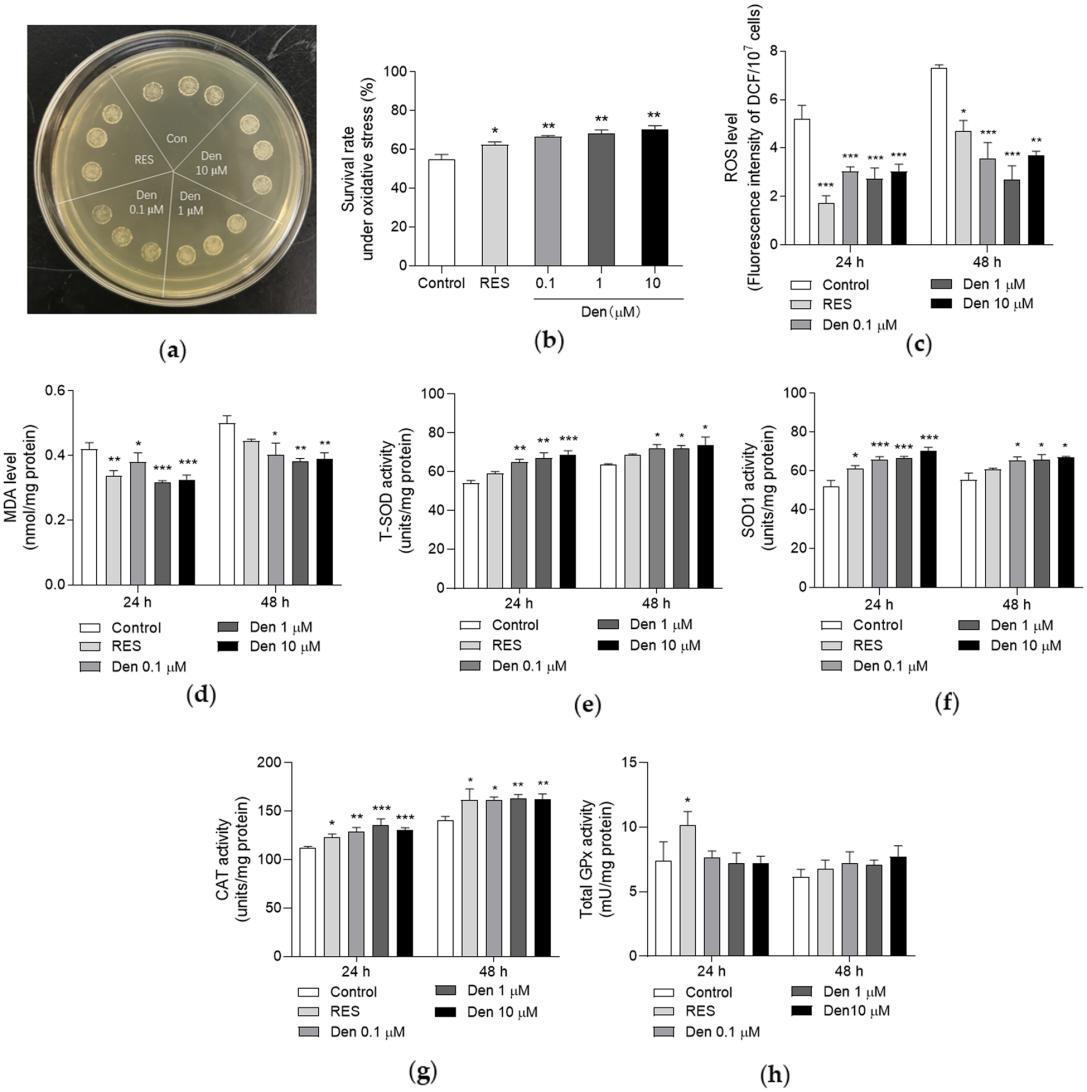
Increased anti-oxidant capability of yeast upon dendrobine treatment. **A** Growth status of BY4741 yeast under oxidative stress induced by 9.5 mM H_2_O_2_; **B** The survival rates of BY4741 yeast under oxidative stress induced by 5.5 mM H_2_O_2_; **C**, **D** The levels of ROS (**C**) and MDA (**D**) in BY4741 yeast upon dendrobine treatment at 24 and 48 h; **E–H** The effect of dendrobine on anti-oxidant enzyme activities in BY4741 yeast at 24 and 48 h. The experiment was repeated three times and data from each experiment are displayed as mean ± SEM. The repeats number of each group was five. ^*^, ^**^, ^***^ represent significant difference compared with negative control (*p* < 0.05, *p* < 0.01, *p* < 0.001)

Oxidative stress is a limiting factor of longevity. H_2_O_2_ is usually utilized to induce oxidative stress condition [11, 12]. As shown in Fig. 2A, the growth status of dendrobine- and RES-treated groups were better than that of negative control under oxidative stress. Quantitative results were obtained by performed another experiment and calculating the survival rate of each group. The results are presented in Fig. 2B, dendrobine at concentrations of 0.1 ,1 and 10 µM significantly increased the survival rate of yeast upon treatment with 5.5 mM H_2_O_2_ (*p* < 0.01, *p* < 0.01 and *p* < 0.01). Therefore, dendrobine enhances the anti-oxidant capacity of yeast under oxidative stress.

Excessive ROS leads to the damage of biological macromolecules because of ROS attack [7] and causes oxidative stress. MDA is the product of lipid peroxidation, and its content is usually utilized as an indicator to reflect oxidative stress [11, 12]. Thereby, we detected the ROS and MDA levels of yeast. As shown in Fig. 2C, D, the contents of ROS and MDA in yeast were significantly decreased by dendrobine at 24 h (Fig. 2C, *p* < 0.001, *p* < 0.001 and *p* < 0.001; Fig. 2D, *p* < 0.05, *p* < 0.001 and *p* < 0.001) and 48 h (Fig. 2C, *p* < 0.001, *p* < 0.001 and *p* < 0.01; Fig. 2D, *p* < 0.05, *p* < 0.01 and *p* < 0.01), respectively. The above results suggest the potential of dendrobine to reduce oxidative stress in yeast.

The mechanism in which denfrobine enhances the anti-oxidant capacity and reduces oxidative stress in yeast was determined by investigating the activities of classic anti-oxidant enzymes. The activities of total SOD in all dendrobine-treated groups were significantly enhanced at 24 h (Fig. 2E, *p* < 0.01, *p* < 0.01 and *p* < 0.001), and similar consequences were observed at 48 h (Fig. 2E, *p* < 0.05, *p* < 0.05 and *p* < 0.05). For SOD1 enzyme, its activity was evidently improved upon dendrobine treatment at 24 h (Fig. 2F, *p* < 0.001, *p* < 0.001 and *p* < 0.001) and 48 h (Fig. 2F, *p* < 0.05, *p* < 0.05 and *p* < 0.05). Simultaneously, dendrobine clearly increased the CAT activity at 24 h (Fig. 2G, *p* < 0.01, *p* < 0.001 and *p* < 0.001) and 48 h (Fig. 2G, *p* < 0.05, *p* < 0.01 and *p* < 0.01). However, the activity of GPx was not influenced by dendrobine treatment at 24 or 48 h, although the yeast in RES-treated group showed increased activity of GPx at 24 h (Fig. 2H).

**Fig. 3:**
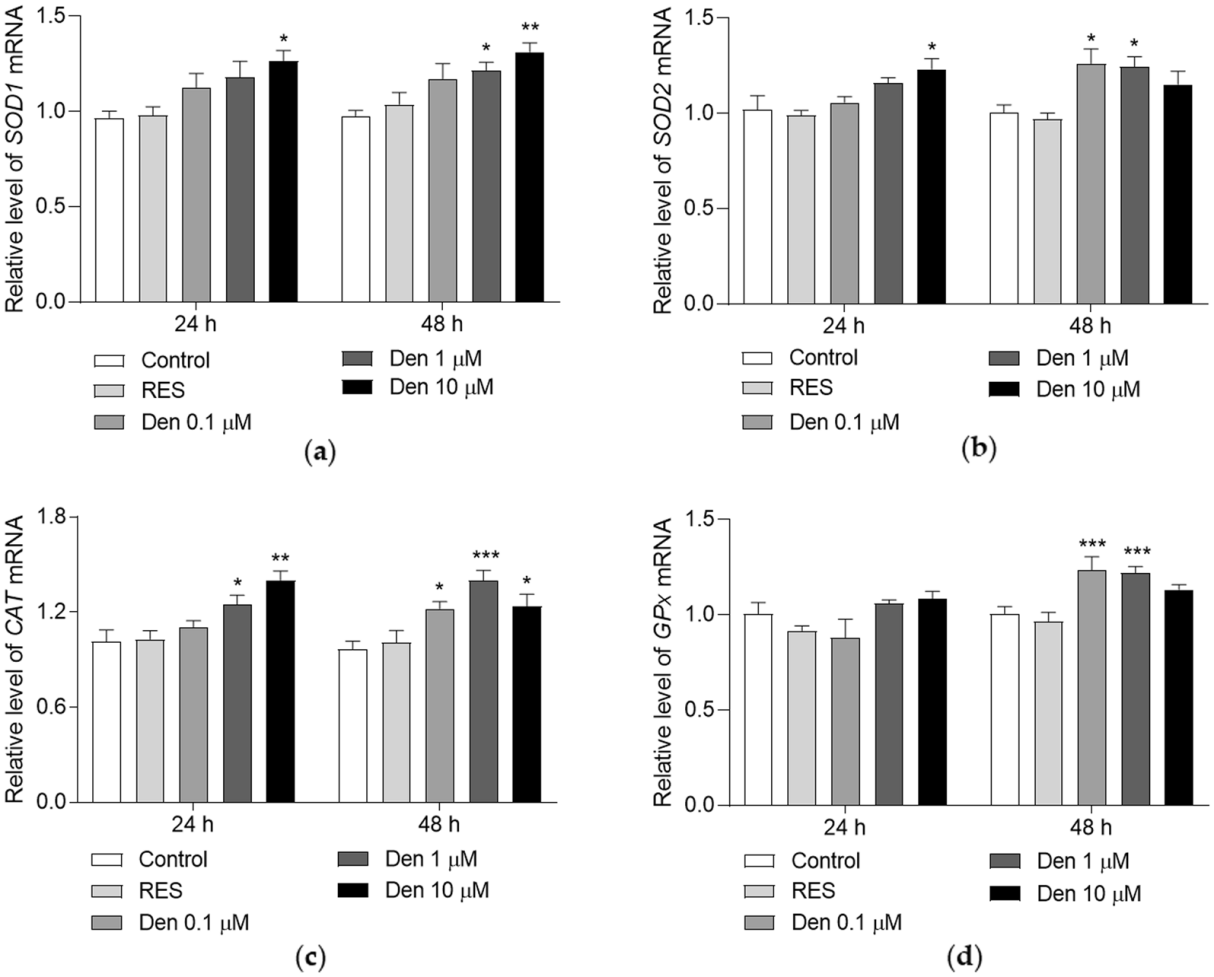
The effect of dendrobine on antioxidant enzyme genes expression. **A–D** The mRNA abundance of *SOD1* (**A**), *SOD2* (**B**), *CAT* (**C**) and *GPx* (**D**) at 24 and 48 h. The experiment was repeated three times and data from each experiment are displayed as mean ± SEM. ^*^, ^**^, ^***^ represent significant difference compared with negative control (*p* < 0.05, *p* < 0.01, *p* < 0.001)

Furthermore, qRT-PCR was used to check the gene expression of above enzymes. The results are displayed in Fig. 3. After treatment with 10 µM dendrobine, the relative level of *SOD1* mRNA significantly increased at 24 and 48 h (Fig. 3A, *p* < 0.05, *p* < 0.01). Dendrobine at a dose of 1 µM significantly enhanced *SOD1* gene expression at 48 h (Fig. 3A, *p* < 0.05). For the *SOD2* gene, the transcription level was evidently improved at 24 h after treatment with 10 µM dendrobine (Fig. 3B, *p* < 0.05), while the mRNA level increased at 48 h after treatment with 0.1 and 1 µM dendrobine (Fig. 3B, *p* < 0.05, *p* < 0.05). The *CAT* gene expression at 24 h was enhanced by dendrobine treatment at doses of 1 and 10 µM (Fig. 3C, *p* < 0.05, *p* < 0.01), and 0.1, 1 and 10 µM dendrobine significantly increased the abundance of *CAT* mRNA at 48 h (Fig. 3C, *p* < 0.05, *p* < 0.001, *p* < 0.05). The mRNA level of *GPx* was not affected by dendrobine at 24 h, but the influence was observed at 48 h after treatment with dendrobine at doses of 0.1 and 1 µM (Fig. 3D, *p* < 0.001, *p* < 0.001).

In summary, dendrobine exerts anti-aging effect by increasing the activities of SOD and CAT, and the increased enzymes activities contribute to the enhanced anti-oxidant capacity and the lower level of oxidative stress in yeast.

### **Involvement of*SOD1***, ***SOD2***, ***CAT***, **and *GPx *genes in the anti-aging effect of dendrobine**

**Fig. 4:**
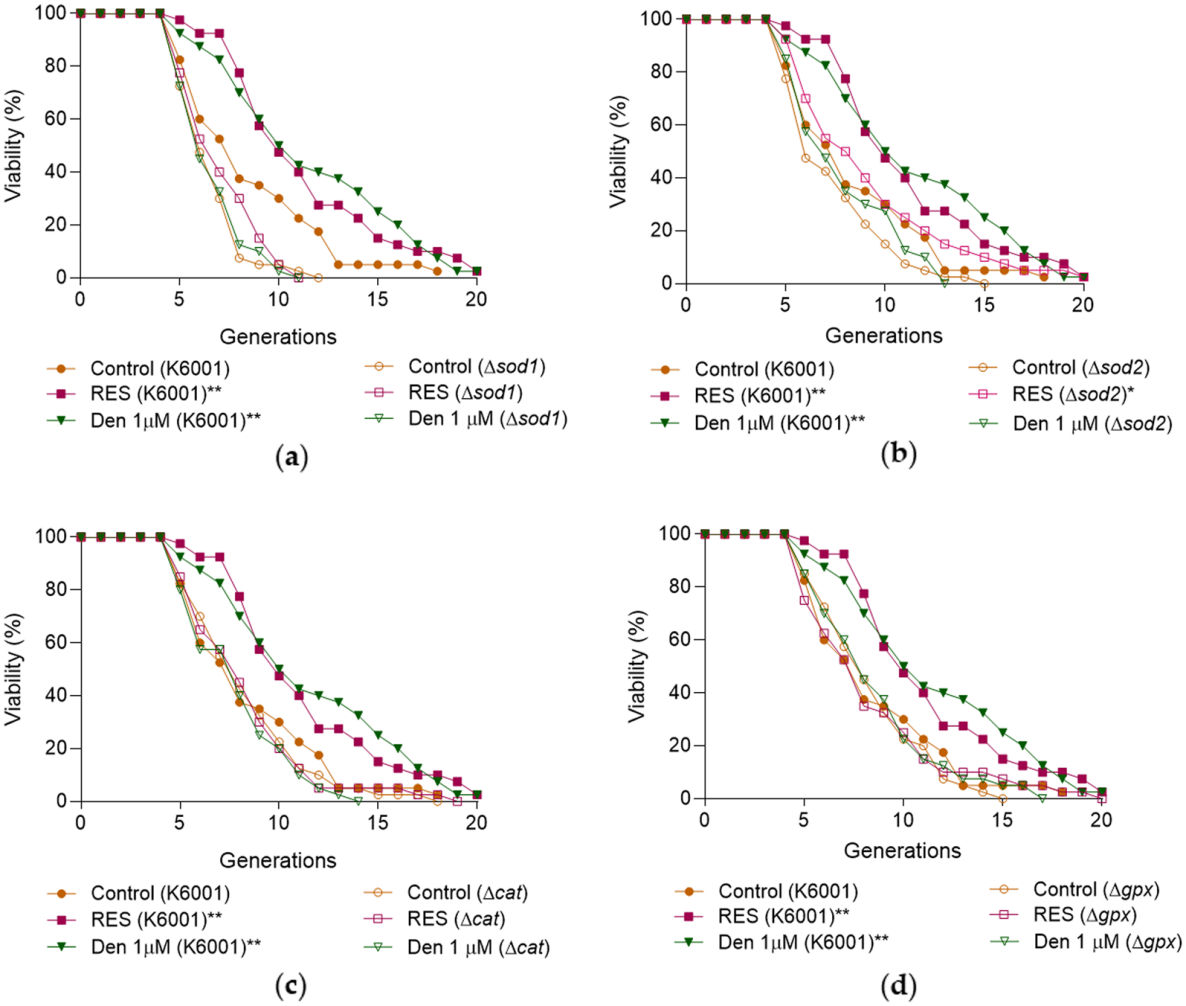
The effects of dendrobine on lifespans of yeast mutants with deletion of antioxidant enzyme genes. **A–D** Dendrobine failed to prolong the replicative lifespans of Δ*sod1* (**A**), Δ*sod2* (**B**), Δ*cat* (**C**) and Δ*gpx* (**D**) yeast mutants. The experiment was repeated three times and data from each experiment are displayed as mean ± SEM. ^*^, ^**^ represent significant difference compared with negative control (*p* < 0.05, *p* < 0.01)

The involvement of *SOD1*, *SOD2*, *CAT*, and *GPx* genes in the anti-aging effect of dendrobine was determined by conducting the assays of replicative lifespans of relevant yeast mutants with K6001 background. Dendrobine extended the replicative lifespan of K6001 yeast but failed to prolong the longevity of Δ*sod1*, Δ*sod2*, Δ*cat*, and Δ*gpx* yeast mutants whose lifespans were close to the corresponding control group (Fig. 4 and Additional file 1: Table S2). These results show that *SOD1*, *SOD2*, *CAT*, and *GPx* genes take part in the anti-aging effect of dendrobine.

### Dendrobine enhances yeast autophagy

**Fig. 5:**
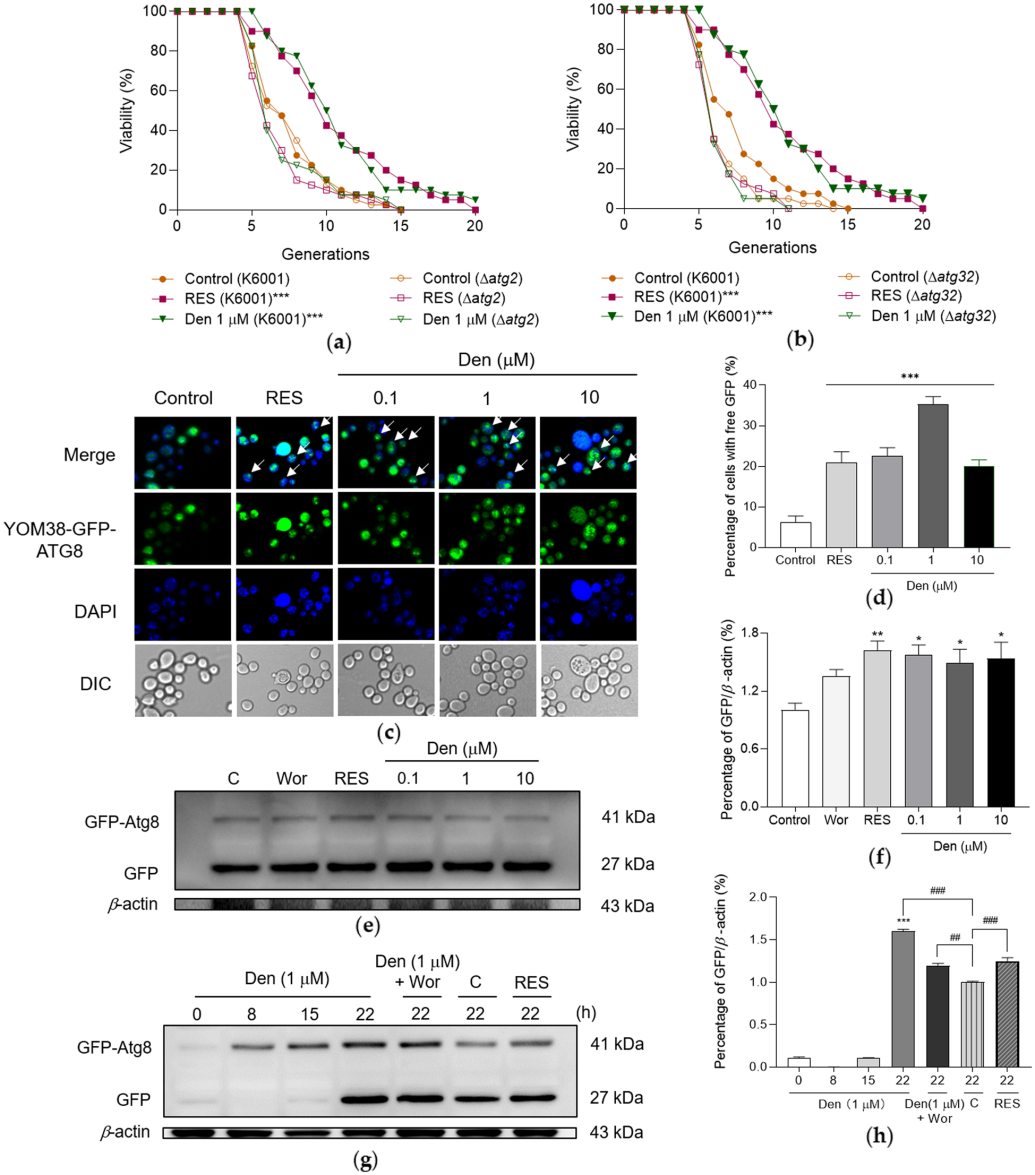
Enhanced autophagy by dendrobine treatment. **A**, **B** Failure of dendrobine to prolong the lifespans of Δ*atg2* and Δ*atg32* yeast mutants with K6001 background. **C** Fluorescent images of yeasts treated with 300 µM RES or 0, 0.1, 1 and 10 µM dendrobine. Punctate green fluorescence is free GFP representing the occurrence of autophagy. **D** Statistical result of **C**; ten pictures of each group were selected randomly, and calculated the percentage of cells with free GFP in each group; ^***^ represents significant difference compared with negative control (*p* < 0.001). **E** The western blot results of GFP-Atg8 and free GFP in yeast after treatment with 200 nM wortmannin (Wor), 300 µM RES and different doses of dendrobine for 22 h. **F** The digital result of **E**, ^*^, ^**^ represent significant difference compared with negative control (*p* < 0.05, *p* < 0.01). **G** The western blot results of GFP-Atg8 and free GFP in yeast after treatment with 300 µM RES, 200 nM wortmannin, 200 nM wortmannin plus 1 µM dendrobine, or 1 µM dendrobine at indicated time. **H** The digital result of **G**; ^***^ represents significant difference compared with dendrobine-treated group at 0 h (*p* < 0.001). ^##^, ^###^ represent significant difference compared with control group at 22 h (*p* < 0.01, *p* < 0.001). The experiment was repeated three times and data from each experiment are displayed as mean ± SEM.

Considering the closely correlation of autophagy and aging, the potential connection between autophagy and the anti-aging effect of dendrobine was determined by conducting the replicative lifespan assays of Δ*atg2* and Δ*atg32* yeast mutants with K6001 background. As presented in Fig. 5A, B, the failure of dendrobine to elongate the lifespans of above mutants was observed, indicating the involvement of autophagy. Next, the autophagic level of yeast upon dendrobine treatment was investigated using YOM38 yeast, which expressed fusion protein GFP-Atg8. The yeasts in dendrobine-treated groups displayed more free GFP (Fig. 5 C, D, *p* < 0.001, *p* < 0.001 and *p* < 0.001), suggesting the induction of autophagy by dendrobine.

Subsequently, Western blot experiment was conducted to verify and quantify the effect of dendrobine on autophagy. Figure 5E, F and Additional file 1:  Fig. S2A show that dendrobine significantly increased autophagy in yeast at doses of 0.1, 1, and 10 µM (*p* < 0.01, *p* < 0.05 and *p* < 0.05), respectively. In addition, the time-course of autophagy was explored upon dendrobine treatment for 0, 8, 15, and 22 h. As indicated in Fig. 5G, H and Additional file 1: Fig. S2B, autophagy began to occur around 15 h of dendrobine treatment and considerably increased at 22 h. Moreover, the autophagy in dendrobine-treated group was not blocked by wortmannin completely, which is an inhibitor of phosphatidylinositol 3-kinase (PtdIns3K). This finding was obtained, because PtdIns3K contributes to autophagosome formation but has no influence in lysosome degradation process [30]. Thereby, the cleavage of fusion protein GFP-ATG8 in lysosome may not be affected by wortmannin. These results confirm that the enhancement of autophagy is an anti-aging mechanism of dendrobine.

### Sch9/Rim15/Msn2 signaling pathway mediates the anti-aging effect of dendrobine

**Fig. 6:**
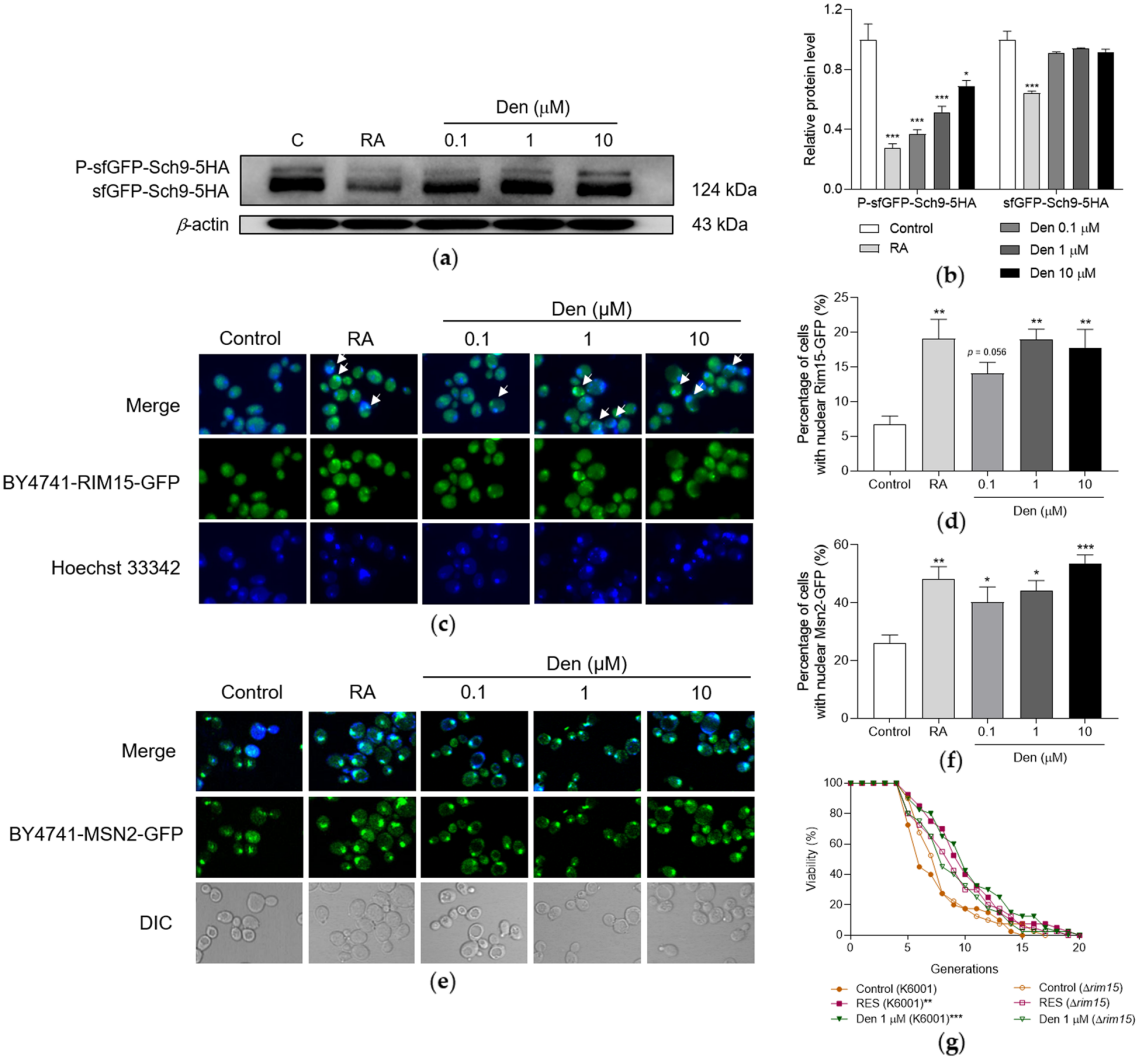
Sch9/Rim15/Msn2 signaling pathway mediates the anti-aging effect of dendrobine. **A** The western blot result of phosphorylation level of sfGFP-Sch9-5HA after treatment with RA or dendrobine for 40 min. **B** The digital result of **A**. **C**, **E** Nuclear translocation of Rim15-GFP (**C**) and Msn2-GFP (**E**) upon dendrobine treatment. **D**, **F** Statistical results of **C** and **E**. Nine images of each group in **C** and five images of each group in **E** were selected randomly, calculated the percentage of cells with the overlap of green and blue fluorescence indicating the nuclear translocation of Rim15-GFP or Msn2-GFP. **G** Failure of dendrobine to extend the replicative lifespan of Δ*rim15* yeast mutant with K6001 background. The experiment was repeated three times and data from each experiment are displayed as mean ± SEM. ^*^, ^**^, ^***^ represent significant difference compared with negative control (*p* < 0.05, *p* < 0.01, *p* < 0.001)

Sch9 is a major target of TORC1 in yeast, and Rim15 is located downstream of Sch9 [18, 21]. When TORC1 is inhibited, Sch9 undergoes dephosphorylation followed by the nuclear translocation of Rim15 [18, 21]. Msn2, which is activated by Rim15 in nucleus, increases the resistance of yeast to oxidative stress by regulating gene transcription [18, 22]. Thus, the Sch9 phosphorylation and nuclear translocation of Rim15 and Msn2 were measured. Dendrobine at doses of 0.1, 1 and 10 µM significantly decreased the abundance of phosphorylated sfGFP-Sch9-5HA (Fig. 6A and Additional file 1: Fig. S3, *p* < 0.001, *p* < 0.001 and *p* < 0.05). In addition, the percentages of cells with nuclear Rim15-GFP (*p* = 0.056, *p* < 0.01 and *p* < 0.01) and Msn2-GFP (*p* < 0.05, *p* < 0.05 and *p* < 0.001) were evidently higher in dendrobine-treated groups than the corresponding control group (Fig. 6 C–F).

The indispensable role of Sch9/Rim15/Msn2 signaling pathway was further confirmed by conducting the replicative lifespan assay of Δ*rim15* yeast mutant with K6001 background. Based on Fig. 6G and Additional file 1: Table S2, no significant difference was observed in replicative lifespans between the control and dendrobine-treated group. These results suggest that dendrobine promotes longevity in yeast through the Sch9/Rim15/Msn2 signaling pathway.

### Interaction between Rim15 and the oxidative stress elimination or autophagy enhancement of dendrobine

**Fig. 7:**
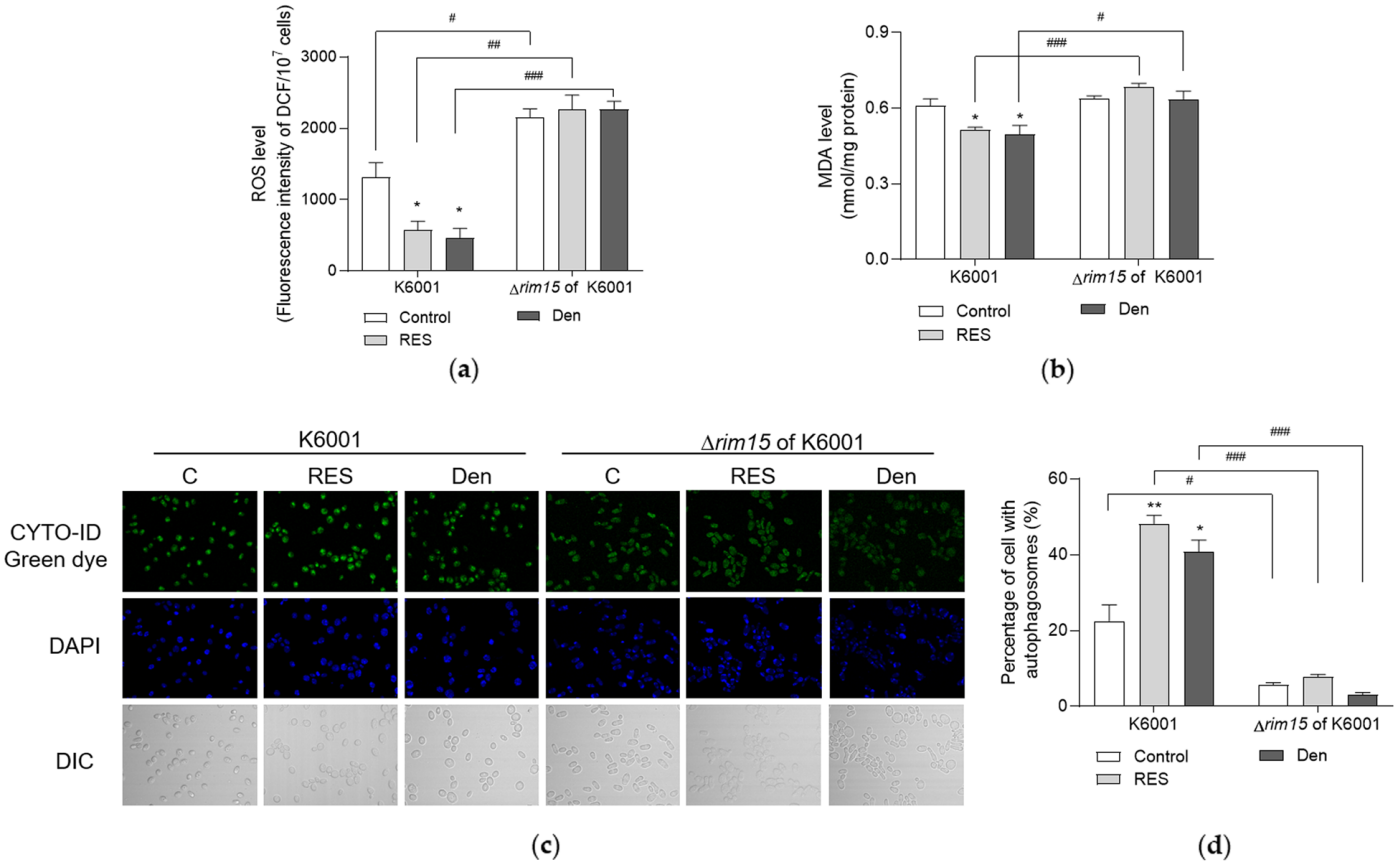
The effects of dendrobine on oxidative stress and autophagy are mediated by Rim15. **A**,** B** Dendrobine failed to decrease the levels of ROS (**A**) and MDA (**B**) in Δ*rim15* yeast mutant with K6001 background. **C** Fluorescence images of autophagy in K6001 yeast and Δ*rim15* yeast mutant with K6001 background after RES or dendrobine treatment. **D** Quantification result of **C**. The experiment was repeated three times and data from each experiment are displayed as mean ± SEM. Ordinary one-way ANOVA followed by Dunnett’s multiple comparisons test were utilized to evaluate statistically significant differences among groups. Two-tailed, unpaired, Student’s t-tests were used for comparison between two groups. ^*^, ^**^ represent significant difference compared with negative control (*p* < 0.05, *p* < 0.01). ^#^, ^##^, ^###^ represent significant difference between K6001 yeast and Δ*rim15* yeast mutant with K6001 background upon corresponding treatment (*p* < 0.05, *p* < 0.01, *p* < 0.001)

Given that the Rim15-dependent transcription of genes involved in oxidative stress response and the nuclear translocation of Rim15 enhances autophagy [23, 24], we speculated that the effects of dendrobine on oxidative stress and autophagy depend on Rim15. Considering the superior performance of dendrobine at a dose of 1 µM on anti-aging effect, this concentration was selected for subsequent experiments. First, the levels of ROS in K6001 and Δ*rim15* of K6001 yeasts were quantified. As shown in Fig. 7A, dendrobine decreased the ROS level in K6001 yeast (*p* < 0.05), whereas this eliminated effect of dendrobine disappeared in Δ*rim15* yeast with K6001 background. The ROS contents of all groups in Δ*rim15* yeast were significantly higher than those in K6001 yeast groups (*p* < 0.05, *p* < 0.01, *p* < 0.001). Moreover, dendrobine was unable to decrease the MDA content in Δ*rim15* yeast with K6001 background, which remained consistent with above ROS results (Fig. 7A, B). Next, autophagic occurrence after dendrobine treatment was examined. Dendrobine enhanced autophagy in K6001 yeast (*p* < 0.05), but failed to increase autophagy in the Δ*rim15* of K6001 yeast manifesting as similar percentages of cells with autophagosomes between control and dendrobine-treated group (Fig. 7 C, D). These results demonstrate that the effects of dendrobine on oxidative stress elimination and autophagy enhancement are mediated by Rim15.

## Discussion

*D. nobile*, as a classical and valuable traditional Chinese medicine, delays aging and prevents aging-related disease in China. The material basis and action mechanism of *D. nobile* to prolong lifespan was determined using the replicative lifespan assay of K6001 yeast as bioactivity system to guide the separation and purification of anti-aging compounds from this Chinese herb. Furthermore, chronological lifespan analysis of YOM36 was used to confirm the anti-aging effect of these compounds, which were separated from this plant. The discovery of dendrobine and the increase in the replicative lifespan and chronological lifespan of yeast after treatment with this compound in Fig. 1 suggest that dendrobine is an essential anti-aging active component of *D. nobile*. This result is consistent with previous report [31].

Oxidative stress is an important factor for aging, and it is caused by excess ROS. ROS can be scavenged by antioxidant enzymes such as SOD and CAT [8]. In addition, autophagy is induced when ROS is excess, which reduce ROS and degrade damage proteins and organelles to ameliorate the side influence of oxidative stress [32]. Dendrobine is incapable of eliminating ROS directly because of the lack of phenolic hydroxyl in the structure, but it still increased the survival rate of yeast under oxidative stress condition and inhibited the contents of ROS and MDA (Fig. 2A–D) in the present study. To clarify the reason why this molecule could resist oxidative stress, we investigated antioxidant enzymes and autophagy. Figures 2, 3 and 5C–H indicate that dendrobine reduces ROS and oxidant damages by increasing antioxidant enzyme activities and autophagy, thereby improving yeast resistance to oxidative stress.

Autophagy is closely related to aging, studies have suggested that it decreased in elderly animal models, and longevity is prolonged by genetic manipulation to increase autophagy [14, 15]. Autophagy is also involved in anti-aging function [11, 12]. Therefore, the changes in the autophagy of YOM38-GFP-ATG8 yeast and lifespans of Δ*atg2* and Δ*atg32* mutant yeasts with K6001 background were detected after treatment with dendrobine. These results in Fig. 5 indicate that dendrobine enhances the autophagy of yeast to extend lifespan.

TORC1 signaling pathway plays an important role for controlling cell growth and metabolism through sensing environmental changes [18]. The inhibition of TORC1 signaling pathway leads to the longevity of yeasts, worms, flies, and rodents [19]. Furthermore, it can regulate oxidative stress via Rim15 and Msn proteins of downstream [18, 21, 23]. Therefore, BY4741-sfGFP-SCH9-5HA, BY4741-RIM15-GFP and BY4741-MSN2-GFP yeast strains were used to detect the activity changes of these proteins after treatment with dendrobine. The reduction of phosphorylated Sch9 and nuclear translocation of Rim15-GFP and Mns2-GFP in Fig. 6 demonstrate that the Sch9/Rim15/Msn2 signaling pathway is involved in the anti-aging effect of dendrobine.

In addition, the Rim15-dependent transcription of genes are  involved in stress response and oxidative stress response [23]. Moreover, Rim15 regulates autophagy through Msn2/4 [18], and the nuclear translocation of Rim15 from cytoplasm leads to the inhibition of Ume6, which induces *ATG8* and then enhances autophagy [24]. Therefore, the interaction between Rim15 and oxidative stress or autophagy was focused on. The results in Figs. 6G and 7 suggest that Rim15 is essential for the regulation of oxidative stress, autophagy, and life extension by dendrobine.

## Conclusion

**Fig. 8:**
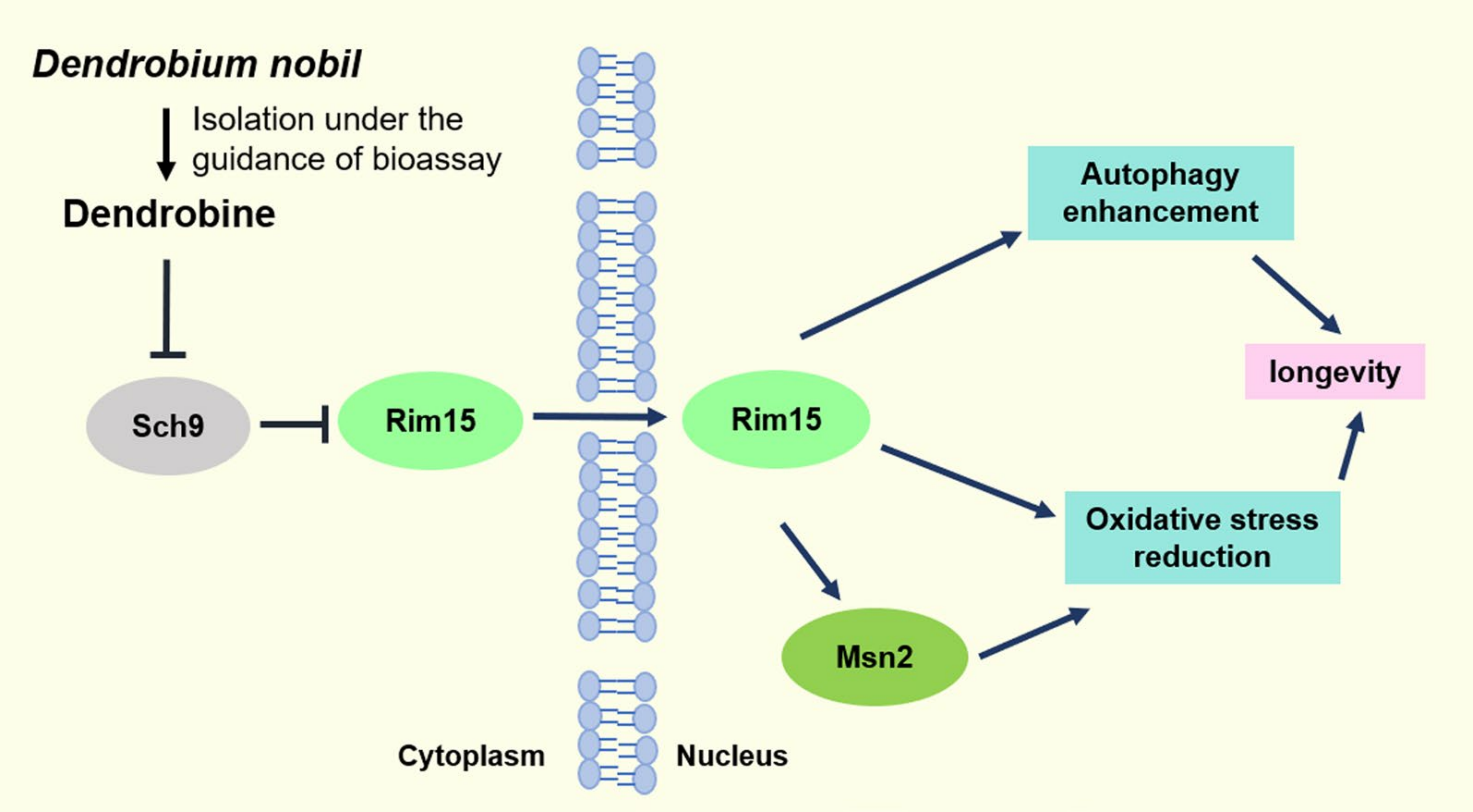
The proposed action mechanism of dendrobine. Dendrobine produced anti-aging effect via inhibiting activity of Sch9 to increase Rim15 activity and Rim15 nuclear translocation to reduce oxidative stress and enhance autophagy via regulation of transcription factor, such as Msn2

Dendrobine from *D. nobile* is an important active component that exerts anti-aging effect in yeast. It prolongs yeast lifespan by inhibiting oxidative stress and enhancing the autophagy mediated by the Sch9/Rim15/Msn2 signaling pathway (Fig. 8). This work laid the foundation for the profundity development of *D. nobile*.

### Supplementary Information

**Additional file 1: Table S1.** Yeast strains used in the present study. **Table S2.** Replicative lifespans of K6001 and its mutants. **Figure S1. **The ^1^H NMR spectrum of dendrobine (500 MHz, CDCl_3_). **Figure S2. **Original data of western blot analysis of free GFP and *β*-actin in Figure 5e and 5g. **a** Original data in Figure 5e present the effect of dendrobine on autophagy. **b** Original data in Figure 5g show the time-course of autophagy upon dendrobine treatment. **Figure S3. **Original data of phosphorylation level of sfGFP-Sch9-5HA in Figure 6a. **a** Original data in Figure 6a show the effect of dendrobine on phosphorylation level of sfGFP-Sch9-5HA.

## Data Availability

The data presented in this study are available from the corresponding author upon reasonable request.
