# Supplementary material for: Involvement of the Sch9/Rim15/Msn2 signaling pathway in the anti-aging activity of dendrobine from Dendrobium nobile Lindl. via modification of oxidative stress and autophagy
Source: Chin Med. 2023 Sep 5;18:111. doi: 10.1186/s13020-023-00827-4 (PMC10481559; doi:10.1186/s13020-023-00827-4)
Supplement: Supplementary file 1 — Additional file 1: Table S1. Yeast strains used in the present study. Table S2. Replicative lifespans of K6001 and its mutants. Figure S1. The 1H NMR spectrum of dendrobine (500 MHz, CDCl3). Figure S2. Original data of western blot analysis of free GFP and β-actin in Figure 5e and 5g. a Original data in Figure 5e present the effect of dendrobine on autophagy. b Original data in Figure 5g show the time-course of autophagy upon dendrobine treatment. Figure S3. Original data of phosphorylation level of sfGFP-Sch9-5HA in Figure 6a. a Original data in Figure 6a show the effect of dendrobine on phosphorylation level of sfGFP-Sch9-5HA. [file 13020_2023_827_MOESM1_ESM.docx]

**Additional Information**

**Sch9/Rim15/Msn2 signaling pathway involves in anti-aging activity of dendrobine from *Dendrobium nobile* Lindl. via modification of oxidative stress and autophagy**

Enchan Wu, Yiting Lian, Sali Zhao, Yajing Li, Lan Xiang* and Jianhua Qi*

College of Pharmaceutical Sciences, Zhejiang University, Yu Hang Tang Road 866, Hangzhou 310058, China

*Correspondence to Lan Xiang and Jianhua Qi.

E-mail addresses: [22019019@zju.edu.cn](mailto:22019019@zju.edu.cn) (E. Wu); [1911689454@qq.com](mailto:1911689454@qq.com) (Y. Lian); [1315797035@qq.com](mailto:1315797035@qq.com) (S. Zhao); [12019045@zju.edu.cn](mailto:12019045@zju.edu.cn) (Y. Li); [lxiang@zju.edu.cn](mailto:lxiang@zju.edu.cn) (L. Xiang); [qijianhua@zju.edu.cn](mailto:qijianhua@zju.edu.cn) (J. Qi).

**1. Additional Method**

Measurement of SOD, CAT and GPx Enzyme Activities

According to the instructions of SOD assay kit (Nanjing Jiancheng Bioengineering Institute, Nanjing, China), 25 µg protein in each group was first mixed with reagent VII and vortexed for 1 min to inactivate the Mn-SOD enzyme in the samples. The supernatant was obtained for the detection of the CuZn-SOD enzyme activity after centrifugation (3000× g, 15 min). The reagent I and blank control samples, and the samples treated by reagent VII were added to the 96-well plate according to the dosage on the instructions. Then, reagents II, III, and IV were added into each well. Then, the plate was incubated at 37 °C for 40 min after mixing well. Finally, the A_550_ absorbance value of the samples was measured after reacting with 200 µL chromogenic working fluid at room temperature for 10 min. The activity of SOD enzyme = ([control group OD value determination group OD value]/control group OD value)/50% × (total volume of reaction solution/sample volume)/protein concentration of sample.

The method of CAT enzyme activity assay was following the manufacturer’s instructions of CAT assay kit (Beyotime Biotech, Shanghai, China). Briefly, gradient concentrations of hydrogen peroxide solution were first prepared. Afterward, chromogenic working fluid was added into the 96-well plate to mix with hydrogen peroxide solution and reacted at 25 °C for 15 min. The standard curve of the hydrogen peroxide concentration was determined after measuring the absorption value at 520 nm. Simultaneously, catalase buffer and 250 mM of hydrogen peroxide were added to each well along with 8 µL protein (1.25 µg/µL). After reacting at 25 °C for 1–5 min, 450 µL enzyme reaction termination solution was added to terminate the reaction. Then, 10 µL of the mixture was taken to react with chromogenic working fluid at 25 °C for 15 min, and the absorption value of A_520_ was measured. The sample catalase activity = [consumption of micromole of hydrogen peroxide] × [dilution ratio]/([reaction minutes] × [sample volume] × [protein concentration]), and [consumed micromole of hydrogen peroxide] = [micromole of residual hydrogen peroxide in blank control] − [micromole of residual hydrogen peroxide of sample].

For the GPx enzyme activity measurement, all the procedures were followed the instructions of GPx assay kit (Beyotime Biotech, Shanghai, China). Almost 5 µg protein of each sample was taken. The general process is that the GPx detection buffer, samples, GPx detection working solution, and peroxide reagent were added to a 96-well plate in turn. The absorbance value of the A_340_ was measured every 4 min, six times, after mixing well. The activity of GPx in the detection system = [(∆A_340_ (sample) − ∆A_340_ (blank))/min]/(0.00622 µM^−1^ cm^−1^ × 0.276 cm). Total GPx activity in the sample = GPx activity in the detection system × dilution ratio/sample protein concentration.

**2. Additional Tables**

**Additional** Table S1. Yeast strains used in the present study

| **Strains** | **Genotype** | **Source** |
| --- | --- | --- |
| K6001 | *MATa*, *ade2-1*, *trp1-1*, *can1-100*, *leu2-3*,*112*, *his3-11*,*15*, *GAL*, *psi*+, *ho*::HO::*CDC6* (at HO), *cdc6*::*hisG*, *ura3*::*URA3* *GAL*-*ubiR*-*CDC6* (at URA3) | Gifted by Professor Michael Breitenbach |
| Δ*sod1*, Δ*sod2*, Δ*cat*, Δ*gp*x, Δ*atg2*, Δ*atg32*, Δ*rim15* of K6001 | Replace the *SOD1* gene, *SOD2* gene, *CAT* gene, *GPx* gene, *ATG2* gene, *ATG32* gene and *RIM15* gene in K6001 with kanamycin gene, respectively | Constructed by Professor Akira Matsuura |
| BY4741 | *MATa*, *his3*Δ*1*, *leu2*Δ*0*, *met15*Δ*0*, *ura3*Δ*0* |  |
| YOM38 containing  pR316-*GFP-ATG8* plasmid | Prototrophic derivative of BY4742 (*MATα*, *his3*Δ*1*, *leu2*Δ*0*, *lys2*Δ*0*) containing plasmid pRS316-*GFP*-*ATG8* |  |
| BY4741 expressing Rim15-GFP | *MATa*, *his3*Δ*1*, *leu2*Δ*0*, *met15*Δ*0*, *ura3*Δ*0;* *RIM15-GFP::His3MX* |  |
| BY4741 expressing Msn2-GFP | *MATa*, *his3*Δ*1*, *leu2*Δ*0*, *met15*Δ*0*, *ura3*Δ*0;* *MSN2-GFP::KanMX* |  |
| BY4741 expressing sfGFP-Sch9-5HA | *MATa*, *his3*Δ*1*, *leu2*Δ*0*, *met15*Δ*0*, *ura3*Δ*0;* *sfGFP-SCH9-5HA::LEU2* |  |

**Additional** Table S2. Replicative lifespans of K6001 and its mutants

| **Figure** | **Yeast strains** | **Treatment (μM)** | **Replicative lifespan (generations)** |
| --- | --- | --- | --- |
| Figure 1(b) | K6001 | Control | 6.95 ± 0.49 |
|  |  | RES 10 | 10.18 ± 0.64^***^ |
|  |  | Den 0.1 | 8.45 ± 0.44^*^ |
|  |  | Den 1 | 9.65 ± 0.55^***^ |
|  |  | Den 10 | 9.10 ± 0.57^**^ |
| Figure 4 (a-d) | K6001 | Control | 7.59 ± 0.57 |
|  |  | RES 10 | 10.45 ± 0.64^**^ |
|  |  | Den 1 | 10.65 ± 0.71^**^ |
|  | Δ*sod1* of K6001 | Control | 5.70 ± 0.26 |
|  |  | RES 10 | 6.20 ± 0.30 |
|  |  | Den 1 | 5.75 ± 0.26 |
|  | Δ*sod2* of K6001 | Control | 6.55 ± 0.41 |
|  |  | RES 10 | 8.45 ± 0.64^*^ |
|  |  | Den 1 | 7.05 ± 0.42 |
|  | Δ*cat* of K6001 | Control | 7.45 ± 0.47 |
|  |  | RES 10 | 7.45 ± 0.49 |
|  |  | Den 1 | 6.98 ± 0.40 |
|  | Δ*gpx* of K6001 | Control | 7.50 ± 0.43 |
|  |  | RES 10 | 7.50 ± 0.59 |
|  |  | Den 1 | 7.72 ± 0.50 |
| Figure 5 (a,b) | K6001 | Control | 6.82 ± 0.42 |
|  |  | RES 10 | 9.64 ± 0.62^***^ |
|  |  | Den 1 | 10.00 ± 0.62^***^ |
|  | Δ*atg2* of K6001 | Control | 6.60 ± 0.40 |
|  |  | RES 10 | 5.98 ± 0.38 |
|  |  | Den 1 | 6.32 ± 0.43 |
|  | Δ*atg32* of K6001 | Control | 5.70 ± 0.30 |
|  |  | RES 10 | 5.55 ± 0.27 |
|  |  | Den 1 | 5.42 ± 0.22 |
| Figure 6 (g) |  | Control | 6.64 ± 0.49 |
|  | K6001 | RES 10 | 9.28 ± 0.58^**^ |
|  |  | Den 1 | 9.55 ± 0.61^***^ |
|  |  | Control | 7.25 ± 0.48 |
|  | Δ*rim15* of K6001 | RES 10 | 8.40 ± 0.59 |
|  |  | Den 1 | 7.87 ± 0.50 |

**3. Additional Figures**

**
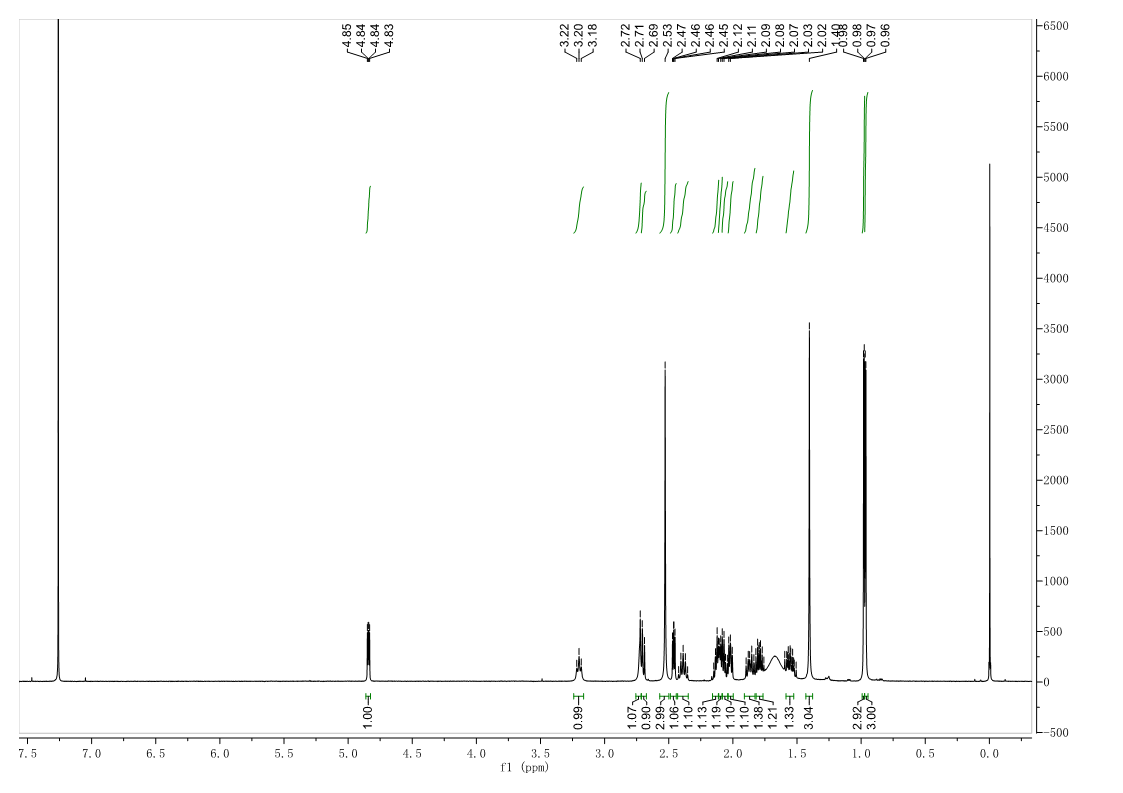
**

**Additional Figure S1:** The ^1^H NMR spectrum of dendrobine (500 MHz, CDCl_3_)

**
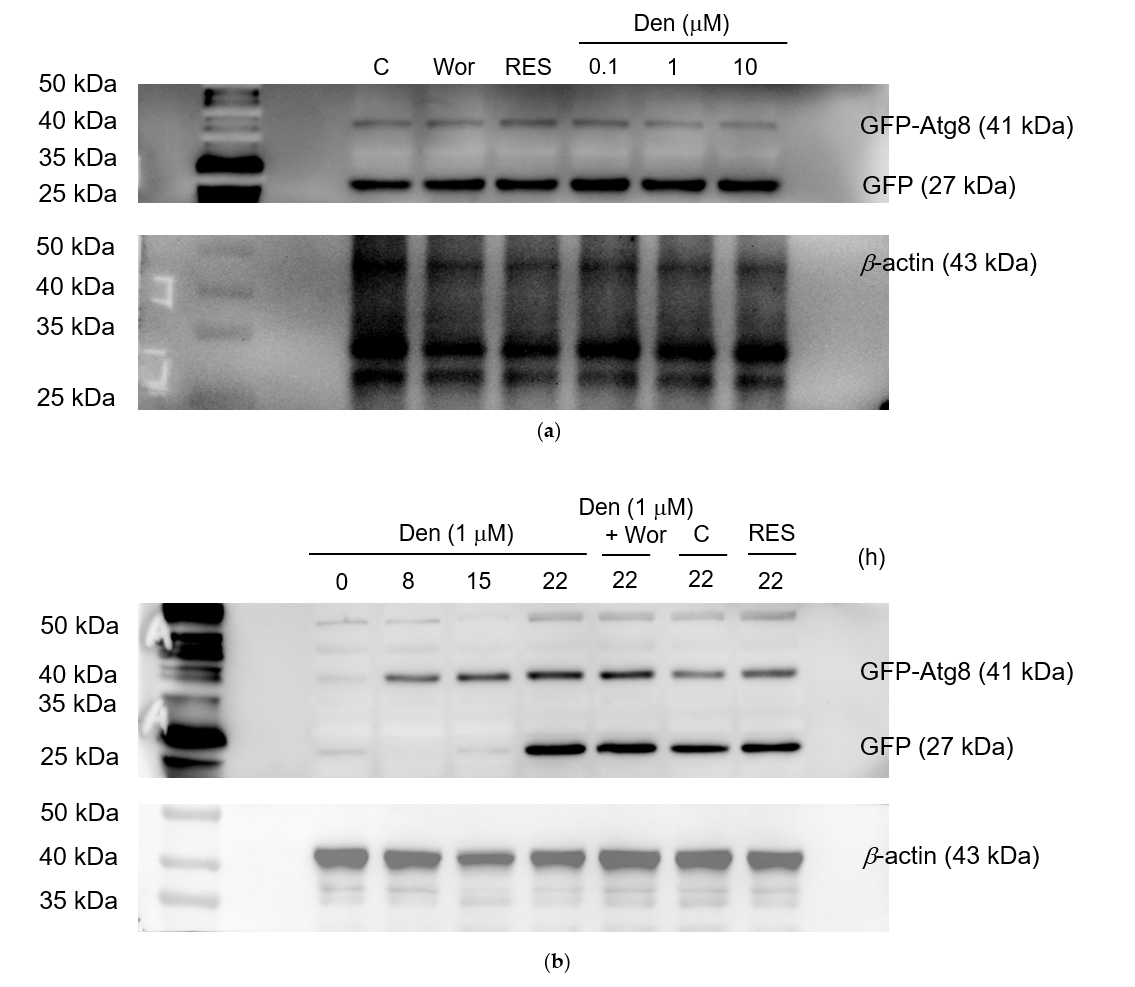
**

**Additional Figure S2:** Original data of western blot analysis of free GFP and *β*-actin in Figure 5e and 5g. (**a**) Original data in Figure 5e present the effect of dendrobine on autophagy. (**b**) Original data in Figure 5g show the time-course of autophagy upon dendrobine treatment.

**
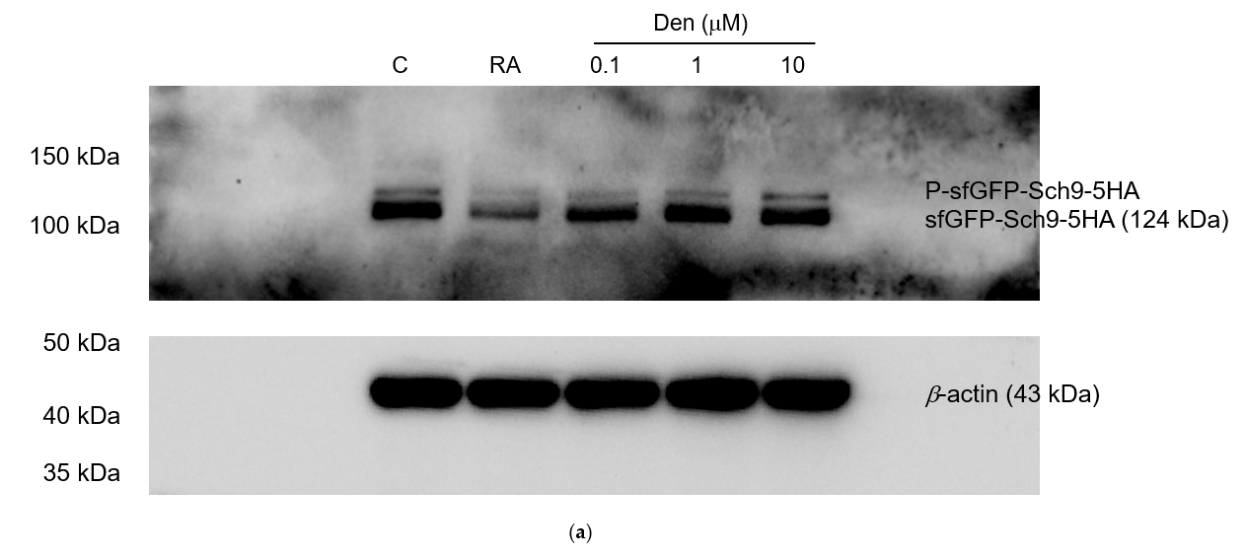
**

**Additional Figure S3:** Original data of phosphorylation level of sfGFP-Sch9-5HA in Figure 6a. (**a**) Original data in Figure 6a show the effect of dendrobine on phosphorylation level of sfGFP-Sch9-5HA.
